# The modified asymmetric chondro-perichondrial island graft in type I tympanoplasty: A retrospective analysis of 784 patients

**DOI:** 10.1016/j.bjorl.2024.101540

**Published:** 2024-12-12

**Authors:** Fetih Furkan Şahin, İsa Kaya, Hakan Ceylan, Tayfun Kirazlı

**Affiliations:** a Kızıltepe State Hospital, Department of Otorhinolaryngology, Mardin, Turkey; b Ege University Faculty of Medicine, Department of Otorhinolaryngology, Izmir, Turkey

**Keywords:** Tympanoplasty, Cartilage, Chronic otitis media

## Abstract

**Objective:**

This study aimed to assess the efficacy of a novel modified asymmetric chondro-perichondrial island graft in tympanoplasty. The design features a longer anterior segment compared to the posterior segment, addressing limitations of symmetrical grafts. We investigated the impact of this modified graft on hearing improvement and graft success rates in patients undergoing tympanoplasty surgery.

**Methods:**

This retrospective study evaluated 784 patients with chronic otitis media who underwent primary type I tympanoplasty with a modified asymmetric chondro-perichondrial island graft technique. Demographics and characteristics of all patients, preoperative location of the perforation, and Pure-Tone Audiometry (PTA) were assessed preoperatively, and graft success and postoperative hearing outcomes were evaluated at the 12-month follow-up.

**Results:**

This study evaluated the efficacy of a modified asymmetric chondro-perichondrial island graft in tympanoplasty. In 784 patients, the mean 12-month postoperative Air-Bone Gap (ABG) improvement was 17.3 dB with a 99% graft success rate. Preoperative ABG significantly improved from 24 dB to 6.6 dB postoperatively (*p* < 0.001). Perforation location did not affect ABG improvement (*p* = 0.193) but did influence graft success rate (*p* < 0.001). No sensorineural hearing loss, retraction pockets, or cholesteatoma were observed postoperatively.

**Conclusion:**

Tympanoplasty offers a well-established surgical approach for restoring hearing function and preventing recurrent otorrhea. The asymmetric cartilage-perichondrium island graft design has emerged as a promising technique to achieve optimal functional and anatomical outcomes in tympanoplasty.

**Level of evidence:**

Level 4.

## Highlights

• Asymmetric graft design accommodates malleus angulation and membrane obliquity.
• Asymmetric design avoids posterior canal contact, suits neutral configuration.
• Larger anterior portion promotes optimal contact with tympanic membrane remnant.
• Asymmetric graft design improves success rate, contributes to better hearing gain.

## Introduction

Tympanoplasty is a well-established surgical technique for repairing perforated Tympanic Membrane (TM) and eradicating chronic otitis media. The primary goals of this procedure are to achieve TM closure with a graft material and restore hearing function.^1^ Tympanoplasty continues to be a mainstay surgical intervention for chronic otitis media, aiming to achieve both eradication of infection and restoration of functional integrity within the tympano-ossicular system. Commonly used graft materials for tympanoplasty include fat, fascia, perichondrium, and cartilage.^2^, ^3^, ^4^, ^5^

In recent decades, cartilage graft techniques have gained significant popularity in tympanoplasty. The ease of harvesting cartilage from the tragus or concha of the auricle, its ability to receive nutrients via diffusion potentially improving graft take rates, and its demonstrated rigidity in the face of non-functional Eustachian tubes and retraction pockets all contribute to its growing use.^6^, ^7^, ^8^ Additionally, promising postoperative hearing outcomes have further supported the expanding adoption of cartilage grafts in tympanoplasty procedures worldwide.^9^

Among cartilage graft techniques for TM reconstruction, the cartilage island graft is particularly common. This technique typically involves removal of a central vertical cartilage strip corresponding to the malleus handle to facilitate its placement. While crucial for accommodating the malleus, this step can impact the creation of the new membrane.^8^ Success in tympanoplasty hinges on understanding both the biomechanics and anatomy of the TM. Notably, the posterior segment of the TM may appear larger than the anterior region based solely on biomechanics. However, the oblique position and adherence of the TM to the annulus and posterior external auditory canal wall complicate this picture.^10^ In underlay technique, cartilage grafts fill the space between the malleus handle and bony annulus. Excessive graft size in this area can lead to stiffness. As a result, a symmetrical notched graft design risks conductive hearing loss due to contact with the posterior canal wall or inadequate coverage of anterior-based or large perforations. Furthermore, contact between the bony annulus and cartilage can limit mobility of both the ossicles and TM. Composite chondro-perichondrial island grafts with peripheral cartilage trimming offer a promising approach to mitigating some of these limitations. Anteriorly located or subtotal TM perforations pose a particular challenge due to the limited availability of anterior TM remnants, which can hinder successful graft placement. Nonetheless, various cartilage tympanoplasty techniques have demonstrated graft success rates of approximately 85%–90% for anterior TM perforation repair.^11^, ^12^

While peripheral cartilage trimming in cartilage island graft techniques is a valuable modification, it does not eliminate all potential complications. Insufficient contact between the graft and the anterior TM remnant can lead to graft dehiscence, whereas excessive contact with the posterior wall can induce blunting and stiffness. Notably, the creation of a central notch for the malleus handle and the formation of symmetrical anterior and posterior portions in the island graft itself can contribute to postoperative audiological complications.

Recognizing these anatomical and functional considerations, the authors of this study developed the modified asymmetric chondro-perichondrial island graft. The novel technique involved removing the cartilage strip for the malleus notch from the posterior half of the graft, resulting in an anterior segment with greater length compared to the posterior segment. This study investigated the impact of a modified asymmetric island graft on hearing outcomes and graft success rates.

## Methods

This retrospective study investigated postoperative 12^th^ month outcomes of the patients who underwent type I tympanoplasty between January 2016 and March 2023. The study was approved by the institutional Research Ethics Committee (Approval number: 20-11.1T/12) and the outcomes of patients were collected retrospectively.

### Study design and participants

A total of 853 patients with chronic otitis media who underwent primary type I tympanoplasty with modified asymmetric island graft technique were screened against inclusion and exclusion criteria. Inclusion criteria comprised dry middle ear cavity with TM perforation with a history of at least 1 year, patients with conductive hearing loss (Air conduction threshold > 20 dB HL) and intact ossicular chain, normal middle ear mucosa, and dry middle ear cavity for at least three months. The exclusion criteria were clinical and radiological evidence of cholesteatoma and chronic mastoiditis, history of prior middle ear surgery on the same ear, history of radiotherapy to the head and neck region. Both pediatric and adult cases were included in the study. Sixty-nine patients were excluded due to loss to follow-up, lack of postoperative examinations, or missing postoperative Pure-Tone Audiometry (PTA) data. Ultimately, 784 patients were enrolled in the study. Preoperatively, TM perforation size, its location, PTA values at frequencies of 500 Hz, 1000 Hz, 2000 Hz, and 4000 Hz were evaluated. Postoperatively, 12^th^ month graft status and PTA outcomes were assessed.

Due to the COVID-19 pandemic, in Turkey, total lockdowns were occasionally implemented between April 2020 and July 2021. Therefore, elective surgeries and postoperative follow-ups could be intermittently performed. With the lifting of the pandemic wide ranging restrictions as of July 2021, all surgeries and postoperative follow-ups were fully performed.

### Surgical technique and instrumentation

Surgical procedures were performed by two experienced otologic surgeons (İK or TK). For endoscopic tympanoplasty procedures, high-resolution recordings were captured using a camera and monitor system (Karl Storz, Germany). The visualization system included a 4 mm diameter, 18 cm long, 0° rigid Hopkins-rod lens endoscope (Karl Storz Endoscopes, Tuttlingen, Germany) illuminated by a Xenon light source (Karl Storz Xenon Nova 175, Tuttlingen, Germany). Microscopic tympanoplasty procedures employed a Carl Zeiss microscope (Oberkochen, Germany).

**Fig. 1:**
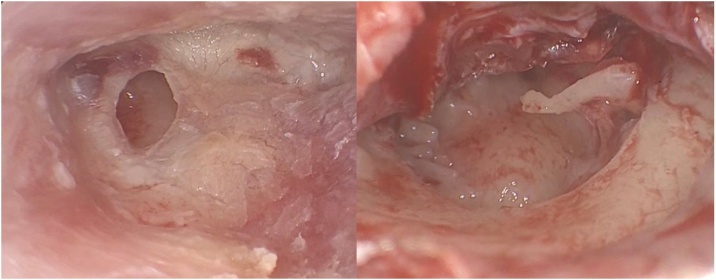
Perforation in the anterior-inferior quadrant of the tympanic membrane, and the view following tympanomeatal flap elevation.

**Fig. 2:**
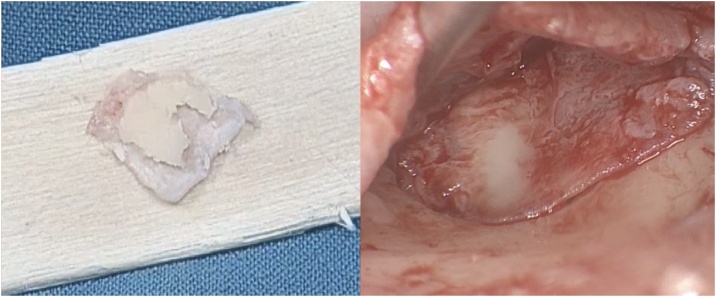
The asymmetric cartilage-perichondrium island graft and placement with over-underlay manner. The graft is sculpted by trimming the cartilage peripherally approximately 2 mm, leaving the perichondrium attached, thinning it to 0.2‒0.5 mm, and creating a notch to accommodate the malleus handle, forming cartilaginous portions with a width of approximately 2‒3 mm posteriorly and approximately 4‒6 mm anteriorly.

First, the perforation edges were deepithelized. After a hemi-circular external auditory canal incision, a tympanomeatal flap was elevated for visualization. The graft material was obtained from the ipsilateral tragal cartilage with the perichondrium on both sides. Perichondrium over the anterior surface of the tragal cartilage graft was removed and the posterior surface was left attached to the cartilage. The surgical technique employed a composite tragal-perichondrial island graft. The cartilage component was thinned to a thickness of 0.2‒0.5 mm. Then, depending on the size and location of the perforation, the graft was prepared by removing a 2 mm vertical strip of cartilage to be compatible with the manubrium mallei, with a width of approximately 2–3 mm posteriorly and approximately 4–6 mm anteriorly, thus, an asymmetric cartilage-perichondrium island graft was formed (Fig. 1). An inadequately sized or shaped notch in the manubrium mallei can compromise graft position. Therefore, creating an appropriately sized notch is crucial for successful graft placement. The graft was positioned using an over-underlay technique, placed lateral to the malleus handle and medial to the tympanic membrane and annulus (Fig. 2). A broad perichondrial attachment, either posteriorly, anteriorly, or anterosuperiorly, can enhance graft stabilization and provide reinforcement in areas of intact tympanic membrane. For instance, a posterior perichondrial attachment extending towards the external auditory canal can both stabilize the graft and promote healing. It is essential to meticulously ensure that the graft is fully seated, conforming to the perforation edges, and that the cartilage components are not medialized or lateralized by not contacting the bone annulus before concluding the surgery. In the final step, the tympanomeatal flap was meticulously repositioned to its original anatomical location, and the external auditory canal was packed with Gelfoam and antibiotic ointment.

### Outcome measures

Preoperative and postoperative 12^th^ month audiological assessments were conducted using standard PTA (Interacoustics AC-40, Middelfart, Denmark) with TDH39 headphones at frequencies of 500 Hz, 1000 Hz, 2000 Hz, and 4000 Hz. Bone Conduction (BC) and Air Conduction (AC) thresholds, and Air-Bone Gap (ABG) levels were calculated at both time points. Additionally, postoperative ABG improvement was determined at the 12-month follow-up. A clinical audiometer meeting International Organization for Standardization (ISO) standards and adhering to American Speech-Language-Hearing Association (ASHA) criteria was used for all audiometric testing. Micro-otoscopic examinations were performed preoperatively and at the 12-month follow-up to evaluate TM perforation size, location of TM perforation, and postoperative graft status. TM perforation localization was classified into four categories: anterior, posterior, central (occupying less than half of the TM surface area), and subtotal. Successful graft take was defined by the absence of perforation, retraction, or lateralization.

### Statistical analysis

For statistical analysis, the SPSS was used (version 22.0; SPSS Inc., Chicago, IL, USA). Levene test was used to evaluate whether the data had homogeneous variance, and Kolmogorov-Smirnov test was used to analyze the distribution pattern. Chi-Square test was used for the comparison of categorical data. Paired samples *t*-test or Wilcoxon signed rank test were used for the comparison of pre- and post-operative outcomes, based on the distribution pattern. Binary logistic regression model was employed to evaluate the impact of perforation size and location, presence of a rheumatological disease, and active tobacco consumption on graft success. Odds ratios were calculated for variables identified as negative risk factors for graft success. Data were expressed as “mean (Standard Deviation; SD)”, percent (%), minimum–maximum, and “median” where appropriate; *p* < 0.05 was considered to be statistically significant.

## Results

This retrospective study recruited 784 patients undergoing type I tympanoplasty with modified asymmetric island graft technique. The patients' ages ranged from 12 to 67 years, with a mean age of 37.8 ± 11.3 years. The gender distribution was 39% female (306 patients) and 61% male (478 patients). Among the 784 patients, 751 (95.7%) were classified as adults, while the remaining 33 (4.3%) were pediatric. A history of active tobacco consumption was identified in 127 patients (16.1%). Rheumatological diseases (including rheumatoid arthritis, systemic lupus erythematosus, Behcet’s disease, and familial Mediterranean fever) were present in 18 patients (2.3%).

Microscopic and endoscopic surgical approaches were employed in 172 (21.9%) and 612 (78%) patients, respectively. Eight patients (1%) required revision surgery within the first postoperative year due to graft failure. Notably, 6 of these revisions were minor procedures (fat, platelet-rich fibrin, or silastic sheet myringoplasty) performed under local anesthesia, while the remaining 2 patients underwent revision cartilage tympanoplasty under general anesthesia. The median follow-up duration was 18 months. Demographics and characteristics of the study population are presented in Table 1.

**Table 1:** Demographics and characteristics of the study population.

Parameter	Number and Rate (%)
Age (mean ± SD)	37.8 ± 11.3
Gender	
Male	478 (61%)
Female	306 (39%)
Rheumatological Disease	
None	766 (97.7)
Present	18 (2.3%)
Other Comorbidities	
None	634 (80.9%)
Diabetes Mellitus	22 (2.8%)
Allergic Rhinitis	89 (11.4%)
Hypertension	23 (2.9%)
Hypothyroidism	16 (2%)
Active Tobacco Consumption	
None	657 (83.8%)
Present	127 (16.2%)
Location of Perforation	
Posterior	307 (39.2%)
Anterior	174 (22.2%)
Central	208 (26.5%)
Subtotal	95 (12.1%)
Surgical Approach	
Microscopic	172 (21.9%)
Endoscopic	612 (78%)
Median Follow-up Duration	18 months

SD, Standard Deviation.

The mean postoperative ABG improvement was 17.3 ± 7.4 dB, and the graft success rate was 99%. Preoperative and postoperative 12-month PTA data and the 12^th^ month graft success rate are presented in Table 2. The mean preoperative ABG averaged across four frequencies was 24 ± 7.6 dB, improving to 6.6 ± 4 dB postoperatively (*p* < 0.001). Statistically significant difference was observed between preoperative and postoperative AC thresholds at all measured frequencies (*p* <  0.001). No statistically significant difference was observed between the preoperative and postoperative BC average thresholds (*p* = 0.377). Comparisons between the mean preoperative and postoperative average PTA values are presented in Table 3.

**Table 2:** Preoperative and postoperative mean pure-tone audiometry outcomes.

Preoperative PTA values					
	500 Hz	1000 Hz	2000 Hz	4000 Hz	Average
BC Thresholds (dB)	9.0 ± 4.2	10.1 ± 4.7	11.6 ± 5.2	13.1 ± 5.5	11 ± 4.4
AC Thresholds (dB)	30.8 ± 7.2	34.4 ± 7.8	33.3 ± 9.2	41.1 ± 9	34.9 ± 7.5
ABG (dB)	21.8 ± 7.6	24.3 ± 7.8	21.7 ± 9.3	28 ± 9.7	24 ± 7.6
Postoperative PTA values					
	**500 Hz**	**1000 Hz**	**2000 Hz**	**4000 Hz**	**Average**
BC Thresholds (dB)	9.1 ± 4.2	9.9 ± 4.8	11.7 ± 4.9	12.9 ± 5.1	10.9 ± 4.4
AC Thresholds (dB)	13.3 ± 4.7	18.8 ± 5.2	15.1 ± 6	22.7 ± 5.7	22.7 ± 5.7
ABG (dB)	4.2 ± 4.1	8.8 ± 4.7	3.5 ± 5.3	9.8 ± 4.9	6.6 ± 4
ABG closure (dB)	17.3 ± 7.4				
Overall graft success rate	99%				

BC, Bone Conduction; AC, Air Conduction; ABG, Air-Bone Gap; SD, Standard Deviation.

**Table 3:** Comparison of the mean preoperative and postoperative pure-tone audiometry average values.

	Preoperative	Postoperative	*p*-value
Bone Conduction (dB)	11 ± 4.4	10.9 ± 4.4	0.377
Air Conduction (dB)	34.9 ± 7.5	22.7 ± 5.7	<0.001
Air-Bone Gap (dB)	24 ± 7.6	6.6 ± 4	<0.001

The study population was grouped according to location of perforation; no statistically significant difference in ABG improvement was observed among the groups in Kruskal Wallis-H Test (*p* = 0.193). However, there was a statistically significant difference in graft success rates among location groups (*p* < 0.001) (Table 4). No patients experienced sensorineural hearing loss, retraction pocket or cholesteatoma development as a postoperative complication during the follow-up period.

**Table 4:** Comparison of postoperative air-bone gap closure levels and graft success rates across perforation location groups (Kruskal-Wallis Test).

Location of Tympanic Membrane Perforation (number of patients)					
	Posterior (307)	Anterior (174)	Central (208)	Subtotal (95)	*p*-value
ABG Closure (dB)	26.2 ± 6.9	26 ± 7.5	25.5 ± 7.9	27.6 ± 8.2	0.193
Graft Success Rate	100%	99.4%	100%	92.6%	<0.001

ABG, Air-Bone Gap.

Binary logistic regression analysis revealed that perforation size (*p* = 0.154), perforation location (*p* = 0.352), and active tobacco consumption (*p* = 0.709) were not significantly associated with morphological graft success. In contrast, the presence of a rheumatological disease was found to have a significant negative impact on graft success (*p* < 0.001, Odds Ratio = 97.821). Of the 18 patients with rheumatological diseases, 5 (27.8%) experienced graft failure at the end of the follow-up period.

## Discussion

This retrospective study evaluated the efficacy of asymmetrically designed cartilage island graft technique. The technique achieved a high success rate of 99% and a statistically significant mean hearing gain of 17.3 ± 7.4 dB at a minimum follow-up of 12 months. Notably, eight patients experienced graft failure. Among these patients, five had a history of rheumatologic disease and another two had diabetes mellitus, suggesting potential risk factors for this complication.

The optimal technique for cartilage tympanoplasty remains controversial, despite the existence of various methods. Consensus is lacking on aspects like ideal graft thickness and the most effective surgical approach. Cartilage palisades and composite cartilage-perichondrial island grafts are commonly employed techniques.^13^ Regardless of perforation size and location, overall cartilage-perichondrium island graft success rate varies in the literature. Bedri et al. reported a 90.3% success rate for the double-layer cartilage-perichondrial island graft plus perichondrium technique, demonstrating a 20.1 dB improvement in average PTA hearing levels.^14^ In a study conducted by Lade et al. comparing endoscopic and microscopic tympanoplasty approaches, overall graft success rate was found to be 83.3%.^15^ El-Hennawi et al. reported a 92.8% graft success rate and a mean ABG improvement of 9.2 ± 5.1 dB by using cartilage-perichondrium island graft in tympanoplasty.^16^ Likewise, Ravi et al. found success rates of 96% and 92% for endoscopic and microscopic cartilage island graft tympanoplasty, respectively.^17^ Similarly, Ayache et al. reported a 96% success rate in a series of 30 endoscopic cartilage island graft tympanoplasties.^18^ In their study, two patients initially presented with residual perforations, which subsequently healed spontaneously.

Lee et al. reported 100% graft integration in island graft cartilage tympanoplasty in 10 patients.^19^ However, 50% of these patients achieved an ABG closure of 50% or greater, indicating a potential disconnect between graft integration and functional hearing improvement. Demirpehlivan et al. investigated the outcomes of two cartilage graft techniques for tympanoplasty.^20^ Their study found a success rate of 97.7% for the cartilage-perichondrium island graft technique in 34 patients, with a postoperative ABG closure of approximately 10 dB. In comparison, the palisade graft technique achieved a success rate of 79% in 19 patients, with a slightly larger postoperative ABG closure of approximately 13 dB. A meta-analysis by Jalali et al. found an overall integration rate of 92% for cartilage grafts.^21^ In this 7-year retrospective study of 784 patients, the asymmetric cartilage-perichondrium island graft yielded a superior clinical outcome with both 99% success rate and 17.3 ± 7.4 dB mean improvement in ABG, potentially surpassing functional and morphological outcomes reported in most prior studies. The significantly higher graft success rate observed in this large patient cohort, compared to previous studies, can be primarily attributed to the optimal anatomical fit of our modified asymmetric cartilage graft design. This design closely mirrors the natural relationship between the tympanic membrane and the manubrium of the malleus. By addressing the limitations of traditional symmetric grafts, such as potential graft medialization from the posterior aspect and inadequate coverage of anterior or subtotal perforations, our modified design ensures comprehensive and secure graft placement. We mainly attribute the significant hearing gain observed in this large patient cohort primarily to the higher graft success rate and meticulous graft placement during surgery. Factors such as graft thickness and material do not appear to differentiate the modified asymmetric cartilage island graft from other cartilage graft types.

Anterior perforations present a significant challenge due to limited surgical access, reduced tissue vascularity, and potential graft instability leading to medialization and residual perforations. Inadequate graft placement and excessive elevation of the annulus can further compromise hearing outcomes.^22^ Reported success rates for anterior perforation repair using various techniques range from 85% to 90%.^11^, ^23^ Mohanty et al. compared cartilage and temporalis fascia grafts in endoscopic type I tympanoplasty for central perforations involving the anterior quadrant.^24^ They reported an overall graft take rate of 91.9%, with a successful ABG closure rate (defined as closure exceeding 50%) of 62.8% and a mean ABG improvement of 17.5 dB in the cartilage island graft group.

Perforation size also influences surgical complexity, particularly when limited tympanic membrane remnants are present, especially anteriorly. Preoperative assessment of perforation size is crucial for selecting the appropriate graft technique and predicting postoperative outcomes. Zhang et al. reported functional and morphological success rates of 85.4% and 89.6%, respectively, at the 12-month follow-up in endoscopic cartilage tympanoplasty for subtotal perforations.^25^ Kazikdas et al. reported a 95.7% graft success rate in a study of 23 patients undergoing palisade cartilage graft technique for subtotal perforation repair. Postoperatively, 14 of these patients (60.9%) achieved an ABG below 15 Db.^26^ Choi et al. performed endoscopic cartilage tympanoplasty for subtotal TM perforations in 239 patients, and reported graft success rate of 86.2% at 6 months postoperatively and mean ABG closure of 10 dB in patients with graft success.^27^ This study revealed no significant differences in ABG improvement observed across perforation locations or sizes. Interestingly, the ABG improvement in both anterior perforations (26 ± 7.5 dB) and subtotal perforations (27.6 ± 8.2 dB) was comparable to the overall hearing gain, further supporting the statistically insignificant influence of perforation location on outcomes. On the other hand, 7 patients who experienced graft failure had subtotal perforation, and the graft success rate for the subtotal perforation group was found to be 92.6%. Among the eight patients with graft failure, only one presented with an anterior perforation. The success rate remained high within the anterior perforation group (99.4%). All perforations categorized as posterior or central achieved a 100% graft success rate in this study. Despite a statistically significant difference in graft success rates observed among perforation locations, the subgroup of 95 patients who underwent type I tympanoplasty for subtotal perforations achieved a noteworthy 92.6% graft success rate. Notably, 6 (6.3%) of these patients had underlying rheumatologic diseases.

Compared to conventional cartilage island grafts, our modified design offers two key advantages. Firstly, this design helps prevent contact between the posterior aspect of the graft and the posterior bony canal wall. Such contact, particularly if caused by a symmetric island graft leaning posteriorly, can lead to loss of sufficient vibration of the neo membrane in this region. Secondly, the larger anterior portion ensures complete coverage of the space anterior to the malleus handle, facilitating healthy contact with the remaining tympanic membrane. The rationale lies in the anatomical features of the TM and malleus handle. Due to the oblique orientation of the TM and the posterior positioning of the ossicular chain, a symmetrical graft may contact the posterior external auditory canal wall, potentially inducing conductive hearing loss. Moreover, the over-underlay tympanoplasty technique necessitates dissection of the malleus handle from the TM to facilitate graft placement over the handle and bony annulus. This dissection can lead to a slight posteromedial shift of the malleus handle towards the incus and stapes due to a loss of stabilizing tension. Consequently, the horizontal axis distance between the malleus handle and the anterior bony annulus may become greater than the posterior distance. Our approach emphasizes the importance of three-dimensional anatomical considerations during graft design. The asymmetry of the island graft, with a smaller posterior section and a larger anterior portion, allows for improved coverage of anterior perforations, particularly in large defects. Therefore, the high graft-take rate and hearing improvement in this study may be attributable to this design, ensuring optimal anatomical compatibility with the tympanic membrane.

This study has several limitations. Firstly, the study was retrospective in design. Secondly, the postoperative assessments were limited to one-year follow-up period and did not allow investigating longer-term outcomes. Another limitation is that the study did not include a comparison analysis between this graft design and other graft techniques. Despite these limitations, the large patient sample size strengthens the study’s findings.

## Conclusion

Cartilage grafts are widely favored in tympanoplasty. However, the natural, obliquely oriented tympanic membrane can present challenges, particularly in achieving coverage of the anterior region. The posterior portion of a conventional graft may protrude against the bony wall, potentially compromising success rates. The asymmetric cartilage-perichondrium island graft design addresses this anatomical hurdle. This study analyzes a large patient cohort undergoing this technique and demonstrates promising results, achieving high success rates in both morphological restoration and hearing improvement. Future prospective, randomized controlled trials are warranted to definitively assess the long-term efficacy of the asymmetric island graft.

## Conflicts of interest

The authors declare no conflicts of interest.
